# Endocrine system involvement in patients with RASopathies: A case series

**DOI:** 10.3389/fendo.2022.1030398

**Published:** 2022-11-18

**Authors:** M. A. Siano, R. Pivonello, M. Salerno, M. Falco, C. Mauro, D. De Brasi, A. Klain, S. Sestito, A. De Luca, V. Pinna, C. Simeoli, D. Concolino, Ciro Gabriele Mainolfi, T. Mannarino, P. Strisciuglio, M. Tartaglia, D. Melis

**Affiliations:** ^1^ Department of Medicine, Surgery and Dentistry “Scuola Medica Salernitana”, Università di Salerno, Salerno, Italy; ^2^ Dipartmento di Medicina Clinica e Chirurgia, Sezione di Endocrinologia, University of Naples “Federico II”, Naples, Italy; ^3^ Dipartimento di Scienze Mediche Traslazionali, Università degli Studi di Napoli “Federico II”, Napoli, Italy; ^4^ Dipartimento di Pediatria, Azienda Ospedaliera di rilievo Nazionale (A.O.R.N). “Santobono-Pausillipon”, Napoli, Italy; ^5^ Dipartimento di Medicina Clinica e Sperimentale, Università “Magna Graecia” di Catanzaro, Catanzaro, Italy; ^6^ Molecular Genetics Unit, Fondazione Casa Sollievo della Sofferenza, Istituto di Ricovero e Cura a Carattere Scientifico (IRCCS), San Giovanni Rotondo, Foggia, Italy; ^7^ Dipartimento di Scienze Biomediche Avanzate, Università degli Studi di Napoli Federico II, Naples, Italy; ^8^ Department of Advanced Biomedical Sciences, University of Naples Federico II, Naples, Italy

**Keywords:** RASopathies, bone mineral density, vitamin D, autoimmunity, CFC

## Abstract

**Background and Objectives:**

Endocrine complications have been described in patients affected by RASopathies but no systematic assessment has been reported. In this study, we investigate the prevalence of endocrine disorders in a consecutive unselected cohort of patients with RASopathies.

**Study Design:**

72 patients with a genetically confirmed RASopathy (Noonan syndrome [NS], N=53; 29 LEOPARD syndrome [LS], N=2; cardiofaciocutaneous syndrome [CFCS], N=14; subjects showing co-occurring pathogenic variants in *PTPN11* and *NF1*, N=3) and an age- and sex-matched healthy controls were included in the study. Endocrine system involvement was investigated by assessing the thyroid function, pubertal development, auxological parameters, adrenal function and bone metabolism.

**Results:**

Short stature was detected in 40% and 64% of the NS and CFCS subcohorts, respectively. Patients showed lower Z-scores at DXA than controls (p<0.05) when considering the entire case load and both NS and CFCS groups. Vitamin D and Calcitonin levels were significantly lower (p< 0.01), Parathormone levels significantly higher (p<0.05) in patients compared to the control group (p<0.05). Patients with lower BMD showed reduced physical activity and joint pain. Finally, anti-TPO antibody levels were significantly higher in patients than in controls when considering the entire case load and both NS and CFCS groups.

**Conclusions:**

The collected data demonstrate a high prevalence of thyroid autoimmunity, confirming an increased risk to develop autoimmune disorders both in NS and CFCS. Reduced BMD, probably associated to reduced physical activity and inflammatory cytokines, also occurs. These findings are expected to have implications for the follow-up and prevention of osteopenia/osteoporosis in both NS and CFCS.

## 1 Introduction

RASopathies are a clinically defined group of genetic syndromes affecting development that are caused by germline mutations in genes encoding signal transducers or regulators of the RAS/mitogen activated protein kinase (MAPK) pathway (1, 2). This family of disorders include Noonan syndrome (NS), LEOPARD syndrome (also known as Noonan syndrome with multiple lentigines, LS), cardiofaciocutaneous syndrome (CFCS), Costello syndrome (CS), Mazzanti syndrome (also known as Noonan syndrome-like disorder with loose anagen hair, NS/LAH), neurofibromatosis type I (NFNS), Legius syndrome and an increasing number of other clinically related conditions (3–5). Most of these disorders share craniofacial dysmorphisms, a wide spectrum of congenital cardiac defects, hypertrophic cardiomyopathy, postnatal reduced growth, cutaneous and musculoskeletal abnormalities, and variable neurocognitive impairment and predisposition to certain cancers (3, 5). While short stature is a well-recognized feature in these patients, endocrine system involvement has rarely been systematically assessed. Possible endocrine complications of NS, such as thyroid disease, gonadal functional impairment and abnormal bone metabolism, have recently been reported (6, 7).

In affected male and female patients prenatal growth is generally normal as well as length at birth. Growth failure often appears during the first year of life (8–10). Mean height is usually at lower limit for age during childhood, and then it usually further declines because of delayed puberty and an attenuated pubertal growth spurt. Bone maturity is usually delayed. Genotype–phenotype correlations have been reported indicating that NS patients with pathogenic PTPN11 and RAF1 variants show more severely impaired growth than those with other genotypes (e.g., pathogenic variants in SOS1) (11). The different molecular role of the individual signal transducers and modulators implicated in these disorders on intracellular signaling possibly underlies this and other genotype-phenotype correlations (12–14).

In NS patients, normal-to-elevated serum GH levels associated with low serum IGF1 levels are found, suggesting occurrence of GH insensitivity at the postreceptor level (15, 16). In children with NS/LAH, short stature is commonly associated to proven GH deficiency (17, 18). In vivo and in vitro studies have shown that upregulated RAS/MAPK signaling is associated with impaired IGF1 production. Moreover, pharmacological inhibition of this pathway also improved growth in other NS mouse models (19–21). However, a direct effect of RAS/MAPK functional upregulation has also been demonstrated at the level of the growth plate and longitudinal bone growth. Indeed, recent studies have revealed an important physiological role for SHP2 in growth plate development (22), and hyperactive SHP2 and BRAF function has been shown to impair chondrocyte differentiation during endochondral bone growth (23–25).

In both sexes, the onset of puberty is usually delayed in NS patients and is associated with a decreased pubertal growth spurt (26–28). Fertility does not seem to be affected in women with NS, while gonadal dysfunction with defective spermatogenesis has been reported in males (29–31), which has been related to cryptorchidism, occurring in up to 80% of NS males (27, 32–34). However, defect in spermatogenesis have been described in male patients with and without cryptorchidism (29, 30, 35), and recent studies documented occurrence of Sertoli cell-specific primary testicular insufficiency (36). Consistently, a critical role of SHP2 in the maintenance of Sertoli cell function has been demonstrated (37). Thyroid dysfunction has been described in many patients with RASopathies, including overt and subliclinal hypothyroidism. Moreover, several studies reported occurrence of thyroid autoantibodies in patients with NS (27, 38–42).

Endocrine complications of NS include possible pathology in bone metabolism. Values of bone mineral density assessed by different techniques (dual-energy radiograph absorptiometry or phalangeal quantitative ultrasound) are decreased in NS, NF1 and CS (43, 44–51), with increased risk of osteoporosis.

Recent studies confirmed that 25(OH)vitD and BMD parameters are reduced in CS, and vitamin D supplementation is not sufficient to restore proper BMD values (52). The impairment of bone metabolism could be the result of up-regulation osteoclast development and function associated with a decreased activity of osteoblasts (22, 53). Indeed, increased levels of bone resorption have been reported in NS patients (54).

The aim of the present study was to perform a systematic study of the involvement of the endocrine system in patients with RASopathies, to establish the timing of follow-up and prevention strategies.

### 1.1 Study design

The current study is a prospective case-control study evaluating the complete baseline endocrine profile, as expression of the hypothalamus-pituitary function, in patients with RASopathies and control subjects. Clinical features suggestive for involvement of any specific endocrine system were recorded. In particular the presence of short stature, delayed growth pattern, delayed or precocious puberty, poor or excessive weight gain, recurrent infections, fatigue, bone and/or joint pain, muscle aches and/or weakness, recurrent infections, and increased tendency to fractures were recorded.

Physical activity and sun exposure were also recorded during the enrolment visit in order to study their contribution on bone impairment.

The clinical and biochemical data were correlated with molecular data including involved gene and specific mutation.

## 2 Patients and methods

### 2.1 Patients

Seventy-two patients affected by RASopathies were enrolled in the study. Subjects were followed at the Pediatric Genetic Section of the University Federico II of Naples (N = 50), Department of Medicine, Surgery and Dentistry “Scuola Medica Salernitana”, Salerno (N = 8), Department of Clinical and Experimental Medicine of the University Magna Graecia of Catanzaro (N = 7) and Santobono Hospital in Naples (N = 7).

The protocol was discussed with each patient (or legal tutor) and signed informed consent was obtained. The whole cohort (44 males, 28 female) included the following RASopathies: NS (N = 53), CFCS (N = 14), LS (N = 2). Three subjects showed co-occurring pathogenic variants in *PTPN11* and *NF1*. The mean age at enrolment was 8.7 years, (range: 2 to 26 years). A control group was also enrolled including 50 apparently healthy subjects (30 males, 20 females) with mean age 9.0 years (range: 0-26). The enrolment was carried out according to the following inclusion criteria: (i) molecularly confirmed clinical diagnosis of RASopathy, (ii) informed consent expression to participate to the study.

Within the NS subcohort, patients were heterozygous for pathogenic variants in *PTPN11* (36/53, 67.9%), *SOS1* (9/53, 16.9%), *RIT1* (4/53, 7.5%), *RAF1* (2/53, 3.7%), and *LZTR1* (2/53, 3.7%). Within the CFCS subcohort, patients were heterozygous for pathogenic variants in *BRAF* (11/14, 78.57%), *MAP2K1* (1/14, 7.14%), *MAP2K2* (1/14, 7.14%), and *KRAS* (1/14, 7.14%) (**Table 1**).

**Table 1:** The study cohort. The different RASopathies are subdivided taking into account the involved disease gene(s).

	*PTPN11*	*SOS1*	*RIT1*	*RAF1*	*BRAF*	*LZTR1^a^*	*KRAS*	*MAP2K1*	*MAP2K2*	*NF1* and *PTPN11*
**Noonan syndrome** (N=53)	36	9	4	2	–	1	–	–	–	–
**cardiofaciocutaneous syndrome** (N=14)	–	–	–	–	11	–	1	1	1	–
**LEOPARD syndrome** (N=2)	2	–	–	–	–	–	–	–	–	–
***Neurofibromatosis-Noonan syndrome*** (N=3)	–	–	–	–	–	–	–	–	–	3

a Dominant condition due to a de novo pathogenic variant.

The diagnosis of NS vs CFCS was performed on the basis of both clinical and molecular data. All the patients carrying *BRAF* mutation received a clinical diagnosis of CFCS.

All patients underwent clinical assessment, including auxological parameters, and patients/families were also contacted to collect available clinical information. Indeed clinical data were available from medical records over the past 20 years. In the medical record a diagnosis of lymphoblastic leukemia was performed in a patient when she was 7; at the time of the study entry the patient was 16. Among NS patients, 8 showed cardiomyopathy. In our patient group, renal abnormalities were reported in the medical record, including double kidney district (1 patient), kidney cyst (1 patient) and pyelectasis (2 patients).

## 3 Methods

### 3.1 Clinical evaluation

We evaluated height and weight, with their percentile and SD scores, BMI, with its percentile and SD scores, growth velocity, and pubertal stage. Short stature was defined when height was at least 2 SD less than the mean for chronologic age. In adults, a BMI between 25 and 30 was considered overweight, whereas, a BMI of 30 was considered obesity. In children, the 85^th^ and 95^th^ percentiles of BMI for age and sex were considered the thresholds for overweight and obesity, respectively. Delayed puberty was defined differently in accordance with sex. In girls, delayed puberty was defined as lack of breast development by 13 years, lack of pubic hair by 14 years, lack of menarche by 16 years, or a the documentation of a period >5 years between thelarche and menarche. In boys, delayed puberty was defined as lack of testicular enlargement by 14 years, lack of pubic hair by 15 years, or the documentation of a period >5 years to complete genital enlargement. Skeletal maturation was evaluated by measuring skeletal age through the examination of a radiograph of the left hand and wrist, according to the method of Greulich and Pyle. Bone mineralization was investigated by dual-energy X-ray absorptiometry (DXA). The presence of bone/joint pain, muscle aches/muscle weakness, recurrent fatigue and increased tendency to fractures was also recorded. Clinical features suggestive for periodontitis and recurrent infections (especially respiratory tract) as well as mood alterations were also investigated. In order to assess the potential contribution of “environmental” factors, on both Calcium metabolism and BMD, physical activity and sun exposure were recorded during the enrolment visit.

Physical activity was evaluated by administering the International Physical Activity Questionnaire (IPAQ) (55). For each patient the time spent experiencing different kind of activities during the previous week was calculated: vigorous-intensity activity (hard physical effort and the patient breathe harder than normal), moderate-intensity activity (moderate physical effort, walking) and the time spent sitting.

Sun exposure was considered low with at least two of the following criteria: no arm and skin exposure during summer months; no sunbathing or holiday in sunny places; no working outdoors. Otherwise, it was deemed sufficient.

### 3.2 Biochemical study

The complete baseline endocrine profile, as expression of the hypothalamus-pituitary function was evaluated in patients with RASopathy and control subjects.

The somatotropic axis was evaluated by analyzing basal serum growth hormone (GH) and insulin-like growth factor 1 (IGF1) *via* chemiluminescence immune assay (CLIA, Diasorin, Liaison XL). In patients with low IGF1, GH stimulation tests (arginine and, if necessary, clonidine) were performed.

The thyrotropic axis function was assessed by analyzing serum thyroid-stimulating hormone (TSH), free triiodothyronine (fT3), free thyroxine (fT4), anti-Tg and anti-TPO (Siemens, Advia Centaur Immunoassay Systems). Anti-r-TSH autoantibodies were assayed by ELISA (TSH Receptor Autoantibody Human ELISA 2. Generation, Biovendor).

The adrenocorticotropic axis function was investigated by analyzing plasma adrenocorticotropic hormone (ACTH), serum cortisol, androstenedione, dehydroepiandrosterone sulphate (DHEA-S), all measured by using automated immunoassays (Siemens, Immulite 2000 XPi). The gonadotropic axis function was investigated by analyzing serum follicle-stimulating hormone (FSH), luteinizing hormone (LH), 17β-estradiol, testosterone levels, by automated immunoassays (Siemens, Immulite 2000 XPi). 17hydroxyprogesteron (17OHP) blood concentration was determined by CLIA (Pantec, IDS ISYS).

Bone metabolism was studied evaluating parathyroid hormone (PTH), calcitonin (56), 25OH vitamin D levels, using immunoassay with chemiluminescence immune assays (Diasorin, Liaison XL). Considering the vitamin D levels, deficiency was defined by 25OHD levels below or equal to 20 ng/ml, while insufficiency was considered by 25OHD levels between 21 and 29 mg/ml (57). Drug intake and fracture history were included in the medical history. Bone mineralization was investigated by dual-energy X-ray absorptiometry (DXA). Osteopenia was defined as the presence of BMD between -1.5 and -2 SD while Osteoporosis was defined as BMD below -2SD.

In a group of 10 patients and 10 controls available for further blood sampling, screening of a panel of inflammatory molecules was performed including PDGF, IL-1b, IL-1ra, IL-2, IL-4, IL-5, IL-6, IL-7, IL-8, IL-9, IL-10, IL-12(p70), IL-13, IL-15, IL-17, Eotaxin, FGF basic, G-CSF, GM-CSF, IFN-g, IP-10, MCP-1(MCAF), MIP-1a, MIP-1b, RANTES, TNF-a, VEGF. The results of these investigations have been previously reported (42).

### 3.3 Statistical analysis

Each numerical variable was expressed as mean +/- SD. Statistical analysis was performed using SPSS package. Differences in studied parameters between patients and controls were analysed using the t test for unpaired data corrected for Fisher exact test. To investigate the presence of an association between severity of phenotype and either DNA mutation or specific gene involved, χ^2^ test was performed.

Differences with P <0.05 were considered to be significant in all instances.

## 4 Results

### 4.1 Clinical evaluation

Within the NS and CFCS subcohorts, short stature was detected in 21/53 (39.6%) and 9/14 (64.3%) patients, respectively. No patient showed obesity or poor weight gain. Pubertal delay was reported in 1/35 NS males and 1/18 NS females. No patient had history of fractures. The presence of bone/joint pain was recorded in 8 NS patients and 1 CFC patient. Recurrent fatigue was recorded in 6 NS patients. No patient showed clinical features suggestive for periodontitis, recurrent infections as well as mood alterations were investigated.

Physical activity was significantly reduced in patients than in controls. Particularly both vigorous-intensity activity (0.24 ± 0.5 hours *vs* 10.25 ± 3, p<0.001), moderate-intensity activity (2.81 ± 0.2 *vs* 6.98 ± 2.5, p=0.012) and walking (2.96 ± 3.6 *vs* 7.2 ± 6.9, p=0.02) appeared significantly reduced. The time spent sitting every day during the previous week was higher in patients than in controls (62.6 ± 46.2 *vs* 32.8 ± 7, p=0.007).

Sun exposure was low in 21/46 (45.6%) patients and in 10/50 (20%) control subjects.

### 4.2 Hormonal studies

#### 4.2.1 GH-IGF1

Two patients, one CFCS and one NS patients showed GH deficiency (GH <8 ng/ml at both arginine and clonidine stimulation test). Both patients had short stature (<-2DS), pathological growth rate and delayed bone age. They have been treated with GH with improvement of growth pattern. No patient who underwent GH treatment showed sign of heart damage or cardiomyopathy at echocardiography. In the remaining patients both baseline GH and IGF1 were in the normal range. No correlation was detected between molecular data and GH deficiency or short stature.

#### 4.2.2 Thyroid disease

Thyroid function was normal in all tested patients and we did not find any clinical signs and symptoms of hypo-hyperthyroidism. No significant difference was recorded between the CFCS and NS groups and controls. Compared to the control population, anti-TPO autoantibody levels were significantly higher both in the whole cohort and in the NS and CFCS subgroup **(** **Tables 2A–C** **)**.

**Table 2A:** Mean of thyroid hormones values and auto-antibodies in all patients and controls.

	Patients (N=54)	Controls (N=54)	P
	Mean ± SD	Mean ± SD	
**TSH**	2.9 ± 1.1	2.8 ± 0.8	0.8
**FT3**	4.32 ± 0.6	3.8 ± 0.5	0.0002
**FT4**	1.5 ± 0.2	1.3 ± 0.2	0.6
**Anti-TG** (N=38)	91.8 ± 29.6	14 ± 5.2	0.05
**Anti-TPO** (N=38)	49.5 ± 65	9.2 ± 3.7	0.0001
**Table 2B. Mean of thyroid hormones values and auto-antibodies in NS patients and controls.**			
	**Patients (N=41)**	**Controls (N=41)**	**P**
	**Mean ± SD**	**Mean ± SD**	
**TSH**	2.9 ± 1.1	2.8 ± 0.7	0.3
**FT3**	4.4 ± 0.7	3.8 ± 0.5	0.001
**FT4**	1.6 ± 0.2	1.3 ± 0.2	0.6
**Anti-TG** (N=38)	106 ± 32	14 ± 5.3	0.15
**Anti-TPO** (N=38)	51 ± 60	8.6 ± 3	0.003
**Table 2C. Mean of thyroid hormone values and auto-antibodies in CFCS patients and controls.**			
	**Patients (N=13)**	**Controls (N=13)**	**P**
	**Mean ± SD**	**Mean ± SD**	
**TSH**	2.1 ± 0.7	2.8 ± 0.7	0.02
**FT3**	3.8 ± 0.3	3.8 ± 0.4	0.6
**FT4**	1.1 ± 0.1	1.3 ± 0.2	0.026
**Anti-TG** (N=38)	15.5 ± 2.0	15 ± 5.2	0.7
**Anti-TPO** (N=38)	37.5 ± 9.8	10 ± 4.2	0.00001

#### 4.2.3 Gonadal function and puberty

Cryptorchidism was found in 21/44 males (47.7%). Others genital malformations were rare. The age of the enrolled patients did not allow to systematically assess gonadal function. No significant differences were observed between patients and controls, when the gonadotropic axis function was tested **(** **Tables 3A–C** **)**.

**Table 3A:** Gonadotropic axis function in all patients and controls.

	Patients (N=33)	Controls (N=33)	P
	Mean ± SD	Mean ± SD	
**FSH**	7.02 ± 11.83	2.76 ± 2.42	0.0203
**LH**	2. 35 ± 4.08	3.5 ± 5.99	0.2644
**17β-estradiol**	58.26 ± 83.73	85.5 ± 65.47	0.1338
**Testosterone**	129.95 ± 141.48	112 ± 133.35	0.5647
**Table 3B. Gonadotropic axis function in NS patients and controls.**			
	**Patients (N=26)**	**Controls (N=26)**	**P**
	**Mean ± SD**	**Mean ± SD**	
**FSH**	7.9 ± 13.3	2.9 ± 2.4	0.0118
**LH**	2.7 ± 4.5	3.8 ± 5.99	0.4486
**17β-estradiol**	58.1 ± 91.1	85.25 ± 65.5	0.1708
**Testosterone**	142.9 ± 147.9	110 ± 133.3	0.3801
**Table 3C. Gonadotropic axis function in CFC patients and controls.**			
	**Patients (N=6)**	**Controls (N=6)**	**P**
	**Mean ± SD**	**Mean ± SD**	
**FSH**	4.5 ± 1.6	2.6 ± 2.5	0.1199
**LH**	1.4 ± 0.9	1.5 ± 1.8	0.400
**17β-estradiol**	82 ± 57.7	84 ± 67.0	0.8372
**Testosterone**	116.5 ± 136.5	126 ± 116.0	0.9281

FSH, follicle-stimulating hormone; LH, luteinizing hormone.

#### 4.2.4 Adrenocorticotropic axis

The adrenocorticotropic axis function was normal. No significant differences were observed between patients and controls, when we compared plasma ACTH, serum cortisol, androstenedione, 17OHP and DHEA-S.

#### 4.2.5 Bone metabolism

None of the enrolled subjects were taking corticosteroids, or any other medication affecting bone metabolism. None of our patients had history of fractures. The presence of bone/joint pain was recorded in 8 NS patients and 1 CFCS patient. In these NS patients BMD was significantly reduced when compared to patients with no pain (-2.49 ± 0.8 *vs* -1.1 ± 0.4; p=0.03). Recurrent fatigue was recorded in 6 NS patients. No patient showed clinical features suggestive for periodontitis, recurrent infections as well as mood alterations were investigated.

Biochemical evaluation for bone turnover was performed. Biochemical parameters revealed calcium and phosphorous levels in the normal range in all tested patients. Compared to the age-and sex matched control population, however, vitamin D serum levels were significantly lower in patients and in the NS and CFCS subgroups **(** **Tables 4A–C** **)**.

**Table 4A:** Calcium and phosphorus metabolism in all patients and controls.

	Patients (N=33)	Controls (N=33)	P
	Mean ± SD	Mean ± SD	
**PTH **	43.6 ± 21.14	33.2 ± 14.02	0.0229
**25 (OH) Vitamine D**	19.15 ± 6.6	38 ± 15.5	<0.0001
**Calcitonine**	4.5 ± 3.7	6.5 ± 1.5	0.0178
**Calcium**	9.8 ± 0.41	9.9 ± 0.48	0.15047
**Phosphorus**	4.4 ± 0.7	5 ± 0.34	<0.0001
**ALP**	167.6 ± 65.2	196.4 ± 52	0.07
**Dexa BMD **	0.61 ± 0.16	0.8 ± 0.2	0.1964
**Z score**	-1.2 ± 1.06	-0.4 ± 0.6	0.006
**Table 4B. Calcium and phosphorus metabolism in NS patients and controls**			
	**Patients (N=26)**	**Controls (N=26)**	**P**
	**Mean ± SD**	**Mean ± SD**	
**PTH **	42.5 ± 21.4	33 ± 13.7	0.0724
**25 (OH) Vitamine D**	20.3 ± 6.2	38.7 ± 12	<0.0001
**Calcitonine**	4.3 ± 3.97	5.8 ± 1.6	0.0770
**Calcium**	9.8 ± 0.4	9.9 ± 0.4	0.2786
**Phosphorus**	4.4 ± 0.64	5.01 ± 0.4	<0.0001
**ALP**	169.5 ± 65.2	213.9 ± 53	0.06
**Dexa BMD **	0.6 ± 0.2	0.8 ± 0.2	0.1702
**Z score**	-1.6 ± 0.9	-0.85 ± 0.4	0.0611
**Table 4C. Calcium and phosphorus metabolism in CFCS patients and controls**			
	**Patients (N=6)**	**Controls (N=6)**	**P**
	**Mean ± DS**	**Mean ± DS**	
**PTH **	48.3 ± 22.01	33.5 ± 17.4	0.106
**25 (OH) Vitamine D**	14.2 ± 7.2	39.6 ± 18.3	0.003
**Calcitonine**	4.31 ± 2.3	8.2 ± 3.1	0.08
**Calcium**	9.7 ± 0.3	9.9 ± 0.3	0.12
**Phosphorus**	4.5 ± 0.8	4.75 ± 0.5	0.11
**ALP**	158.9 ± 68.03	178.7 ± 51.0	0.08
**Dexa BMD **	0.5 ± 0.2	0.7 ± 0.2	0.0556
**Z score**	-0. 9 ± 0.2	-0.3 ± 0.7	0.0623

ALP, alkaline phosphatase; BMD, bone mineral density; PTH, parathyroid hormone.

PTH serum levels were significantly higher in patients than in controls while calcitonin serum levels were significantly lower. Patients showed lower Z-scores at DXA than controls **(** **Table 4A** **)**. BMD z-scores correlated with physical activity. The time spent sitting every day inversely correlated with BMD z-score levels (r= -0.638, p=0.008). No correlation between BMD data and short stature and/or sun exposure was recorded. It is noteworthy that 4 patients with low BMD showed high cytokines levels (data not shown). No correlation was detected between molecular data and BMD was recorded.

## 5 Discussion

Endocrine system involvement, such as thyroid disease, gonads impairment and abnormal bone metabolism, have been reported in NS (6). The present study provide a general picture of the endocrine system involvement in patients with RASopathies. In particular, this report offers an endocrine system profiling of an unselected CFCS cohort.

As expected, the collected data underlined a high prevalence of short stature both in NS and in CFCS group (58, 59). The reduced growth in both disorders likely results from the dysregulation of the RAS-MAPK signaling pathways at different levels, being related to GH insensitivity at the postreceptor level (15, 16), and to a direct effect at the growth plate (22, 24). While GH deficiency was demonstrated in 1 NS patient and 1 CFCS patient, none of our patients showed a GH insensitivity. No correlation between genotype and short stature was observed.

Thyroid dysfunction has been described in patients with RASopathies, including overt and subclinal hypothyroidism. Moreover, several studies reported occurrence of thyroid autoantibodies in patients with NS (27, 38–42). Our findings outline the high prevalence of thyroid autoimmunity in both NS and CFCS. These data confirm our previous report indicating a high risk to develop autoimmune disease in these groups of patients. Of note, the presence of thyroid autoantibodies was associated to normal thyroid function, in line with previously reported studies (27, 34, 38–41). We hypothesize that these antibodies can precede the clinical symptoms of the disease by years, and could be used for diagnostic and prognostic purposes (42).

Although osteopenia/osteoporosis have anecdotally been reported in NS, pathophysiology and correlations with underlying genetic defects are poorly understood. Reduced BMD in presence of normal bone mineralization marker has previously been reported in NS (44). Low 25-OH vitamin D concentration has been documented in NF1 patients (60, 61) and circumstantially in other RASopathies. However, no clear correlation between vitamin D levels and BMD was reported (45). In the current study, analysis of bone metabolism documented a lower BMD in patients (including both NS and CFCS subgroups) compared to controls. Vitamin D serum levels were significantly reduced, PTH levels significantly increased and calcitonin serum levels reduced in patients than in controls when the entire case load was considered. The observation of decreased levels of calcitonin, marker of osteoblastic activity and bone neo-formation in these patients might be considered as a compensatory mechanism against the increased resorption, which is in turn pointed out by high levels of PTH.

Both NS and CFCS patients also showed lower vitamin D serum levels than controls. However no correlation between vitamin D levels and BMD was demonstrated, suggesting that other factors might cause bone damage in patients affected by RASopathy. The impairment of bone metabolism could be the result of up-regulation of osteoclast development and function (22, 53) with a decreased activity of osteoblasts by the action of SHP2. Increased levels of a bone resorption marker (degraded cross links of mature collagen excreted in the urine) have been reported in NS patients (54), confirming an important role of osteoclasts. Several studies underline that the Ras-MAPK signal transduction pathway is important in bone homeostasis (54, 62, 63), suggesting that increased signaling through this cascade impacts bone remodeling (64, 65). Our results support the hypothesis that metabolic bone disease due to variation in osteoclast vs osteoblast activity as a consequence of a dysregulation of the RAS MAPK signaling pathway is at the basis of impaired bone metabolism (43). However, additional factors that could contribute to the increased bone resorption markers (e.g., inactivity, hypotonia, and poor motor function) should be considered. Consistently, recent study confirms that a combination of different factors, including reduced sun exposure, possibly associated with reduced serum vitamin D levels, and poor physical activity, concur to the impaired bone status in NF1 patients (61).

In this respect it is noteworthy that we demonstrated a correlation between physical activity and BMD data. We also underline that patients with lower BMD show high levels of inflammatory cytokines and high prevalence of joint pain. On the basis of the current data we suggest to investigate bone metabolism and BMD in patients complaining joint pain.

Endocrine imbalance may also be involved in bone abnormalities. The anabolic effect of IGF1 on the bone is well known, regulating bone growth and enhancing osteoblast proliferation. Recently, it has been shown that IGF1 promotes osteoblastic activity activating mTOR pathway. IGF1 affects bone status also acting in a paracrine way, in response to mechanical load, such as during physical activity. It might be hypothesized that low IGF1 levels due to GH resistance might contribute to reduced BMD in both NS and CFCS patients. Furthermore, reduced physical activity could also contribute to the reduction of the DXA z-score in patients as the expression of IGF-1 is stimulated in response to mechanical load.

On the basis of the collected data, we suggest that that the combination of several factor including the dysregulation of the RAS-MAPK pathway, the reduced physical activity, the presence of inflammatory cytokines and the impaired IGF1 activity might contribute to the impaired bone metabolism in patients with RASopathy.

We recommended routine monitoring of bone homeostasis to prevent bone deterioration and possible fractures in both NS and CFCS patients. Regular physical activity should also be encouraged in patients affected by RASopathy when allowed by heart involvement.

## 6 Limits of study

The main limitation of this study is the relatively small number of enrolled patients. Further studies are necessary to investigate the effects of other factors influencing BMD in patients. Longitudinal studies are needed to evaluate the progression of endocrine involvement in patients with NS and CFCS.

## 7 Conclusion

Endocrine complications have been described in patients with RASopathies, though an evaluation of the endocrine system status has not systematically been performed. The collected data demonstrate a high prevalence of thyroid autoimmunity, confirming an increased risk to develop autoimmune disorders both in NS and CFCS. Reduced BMD, probably associated to reduced physical activity and inflammatory cytokines, also occurs. These findings are expected to have implications for the follow-up and prevention of osteopenia/osteoporosis in both NS and CFCS. In order to recommend systematic evaluation of all endocrinological aspects in patients with asymptomatic RASopathy, continuous monitoring will be required and other studies are necessary to confirm our results.

## Data availability statement

The raw data supporting the conclusions of this article will be made available by the authors, without undue reservation.

## Ethics statement

The studies involving human participants were reviewed and approved by Comitato Etico Campania Sud. Written informed consent to participate in this study was provided by the participants’ legal guardian/next of kin.

## Author contributions

Conceptualization, DM, MAS, RP. Methodology, AL, VP, CM, TM. software MAS, AL, VP, CGM, TM. Validation, DM, CS, AL, VP, CGM, TM. Formal analysis MAS, CS, MF, AL, VP, CGM, TM. Investigation CS, CM, AK, SS. Data curation, DM, RP, MS, MF, CM, DB, AK, SS, DC. Writing—original draft preparation, MAS, MF, CM. writing—review and editing. DM, TM, RP, DC, PS. Visualization, DM. supervision, DM, MT, PS, RP. All authors contributed to the article and approved the submitted version.

## Conflict of interest

The authors declare that the research was conducted in the absence of any commercial or financial relationships that could be construed as a potential conflict of interest.

## Publisher’s note

All claims expressed in this article are solely those of the authors and do not necessarily represent those of their affiliated organizations, or those of the publisher, the editors and the reviewers. Any product that may be evaluated in this article, or claim that may be made by its manufacturer, is not guaranteed or endorsed by the publisher.
